# Editorial: Molecular Mechanisms of Pathogen-Driven Infectious and Neoplastic Diseases

**DOI:** 10.3389/fcell.2021.696152

**Published:** 2021-05-31

**Authors:** Giulia De Falco, Davide Gibellini, Pier Paolo Piccaluga, Paul G. Murray, Sam Mbulaiteye

**Affiliations:** ^1^Nanchang Joint Programme, School of Biological and Chemical Sciences, Queen Mary University of London, London, United Kingdom; ^2^Microbiology Section, Department of Diagnostics and Public Health, University of Verona, Verona, Italy; ^3^Department of Experimental Diagnostic and Specialty Medicine, University of Bologna, Bologna, Italy; ^4^Institute of Immunology and Immunotherapy, University of Birmingham, Birmingham, United Kingdom; ^5^Division of Cancer Epidemiology & Genetics Infections and Immunoepidemiology Branch, National Cancer Institute, Bethesda, MD, United States

**Keywords:** pathogens, infectious diseases, cancer, molecular mechanisms, viruses

I would like to start this Editorial by quoting a couple of sentences from the article “Multiple Network-Based Approaches to Identify a Key Regulator of Non-lethal Infections” by Mitchell et al. published in this Research Topic. “*Viruses that are newly introduced to the human population have the potential to be highly pathogenic. While the pathogenicity of these new strains tends to wane as adaptation progresses, emerging viruses are an ever-present threat to human health and the global economy because it is difficult to predict when a new pathogenic strain will appear.”*

When we started discussing about editing a special issue to highlight how pathogens may not only cause infectious diseases, but also contribute to cancer, we would have never imagined that, shortly after, a new pathogen, COVID-19, would emerge, causing a pandemic of biblical proportion and resulting in so many deaths and much grief. COVID-19 quickly became the most intensively studied pathogen as the scientific community worldwide raced to discover effective preventive and therapeutic measures. Three articles published in this Research Topic focus on this new coronavirus and highlight its mechanism of infection (“The mechanisms and animal models of SARS-CoV-2 infection,” by Jia et al.), neurological complications observed in infected patients (“Severe Acute Respiratory Syndrome Coronavirus 2-Induced Neurological Complications,” by Yu and Yu), and explore the possible use of curcumin to counteract the cytokine storm induced by this virus, which seems to contribute to the severity of signs and symptoms experienced by infected individuals (“The Inhibitory Effect of Curcumin on Virus-Induced Cytokine Storm and Its Potential Use in the Associated Severe Pneumonia,” by Liu and Ying). However, inflammation is a key response to all pathogens and, if aberrantly activated, can be harmful and predispose to severe diseases. This crucial aspect is investigated in “Microbes as Master Immunomodulators: Immunopathology, Cancer and Personalized Immunotherapies,” by Lérias et al. in which the association of certain human leukocyte antigen (HLA) alleles with a strong pro-inflammatory response and increased susceptibility to development of specific cancer types in the settings of a pathogenic infection is nicely unraveled. The role of an excessive inflammatory response is further investigated in “LncRNA MALAT1 Affects Mycoplasma pneumoniae Pneumonia *via* NF-κB Regulation,” by Gu et al. in which the Authors illustrate how down-regulation of the lncRNA MALAT-1, which is linked with an excessive inflammatory response, may reduce pulmonary inflammation caused by *Mycoplasma pneumoniae*.

Two articles of this Research Topic focus on Hepatitis B virus and its link with liver dysfunction and hepatocellular carcinoma. In “Hepatitis B e Antigen Induces NKG2A+ Natural Killer Cell Dysfunction *via* Regulatory T Cell-Derived Interleukin 10 in Chronic Hepatitis B Virus Infection,” by Ma et al. the Authors illustrate how the virus increases production of IL-10, which in turn determines an increased expression of NKG2A on NK cells, which is responsible for NK cell dysfunction during chronic hepatitis B infection. Based on their findings, the Authors conclude that HBeAg-IL-10-NKG2A+ NK cell axis is a potential therapeutic target in HBV chronically-infected patients. The link between cytokine release and chronic hepatitis B infection is further unraveled in “IL-35: A Novel Immunomodulator in Hepatitis B Virus-Related Liver Diseases,” by Li et al. In this article the Authors give an overview about how this recently discovered cytokine is key to determine progression from a chronic hepatitis B infection to development of hepatocellular carcinoma and metastatisation. In particular, the Authors illustrate how IL-35 can contribute to this process by inhibiting proliferation and cytotoxicity of HBV-specific cytotoxic T lymphocyte (CTL), deactivating the immature effector T-cells (Teffs), and delaying the proliferation of dendritic cells.

In “Genome-Scale Metabolic Modeling for Unraveling Molecular Mechanisms of High Threat Pathogens,” by Sertbas and Ulgen, the Authors provide an overview about how genome-scale metabolic models are useful to unravel pathogenetic mechanisms used by specific pathogens to infect a host, and how the subsequent interplay develops. Understanding the mechanisms that are involved in this cross-talk and what pathways become unbalanced following an infection may be crucial to design promising therapeutic applications against pathogens for global preventative healthcare.

The application of these sophisticated techniques is further explored in two other articles of this Research Topic, “The Role of EGFR in Influenza Pathogenicity: Multiple Network-Based Approaches to Identify a Key Regulator of Non-lethal Infections,” by Mitchell et al. in which the Authors focus on how the expression of specific genes can be unraveled by network clustering and topology. In this study the Authors highlight that signaling downstream of EGFR, coagulation pathways, and B-cell down-regulation in the lung are tied to infection severity in highly pathogenic influenza strains.

In “Chlorogenic Acid Promotes Autophagy and Alleviates *Salmonella Typhimurium* Infection Through the lncRNAGAS5/miR-23a/PTEN Axis and the p38 MAPK Pathway,” by Tan et al. RNA Seq profiling was used to investigate how lncRNAs are regulated upon immune cell stimulation or pathogen invasion, focusing in particular on lncRNA GAS5. The Authors explore the mechanisms underlying the therapeutic effects of chlorogenic acid in *Salmonella typhimurium* infection, and demonstrate that this treatment up-regulates lncRNA GAS5 in epithelial cells, thus leading to autophagy *via* the miR-23a/PTEN/p38MAPK axis.

The link between Human Papillomavirus infection and cancer is explored in “The Role of RNA Splicing Factors in Cancer: Regulation of Viral and Human Gene Expression in Human Papillomavirus-Related Cervical Cancer,” by Cerasuolo et al. in which the Authors highlight how HPV contributes to the dysregulation of splicing regulation thus leading to the aberrant production of oncogenes. They focus in particular on the role of hnRNP and SR splicing factors in the production of viral oncoprotein isoforms involved in viral life cycle and in cell transformation. Figure 1 summarizes key events that might contribute to virally-driven cancers.

**Figure 1:**
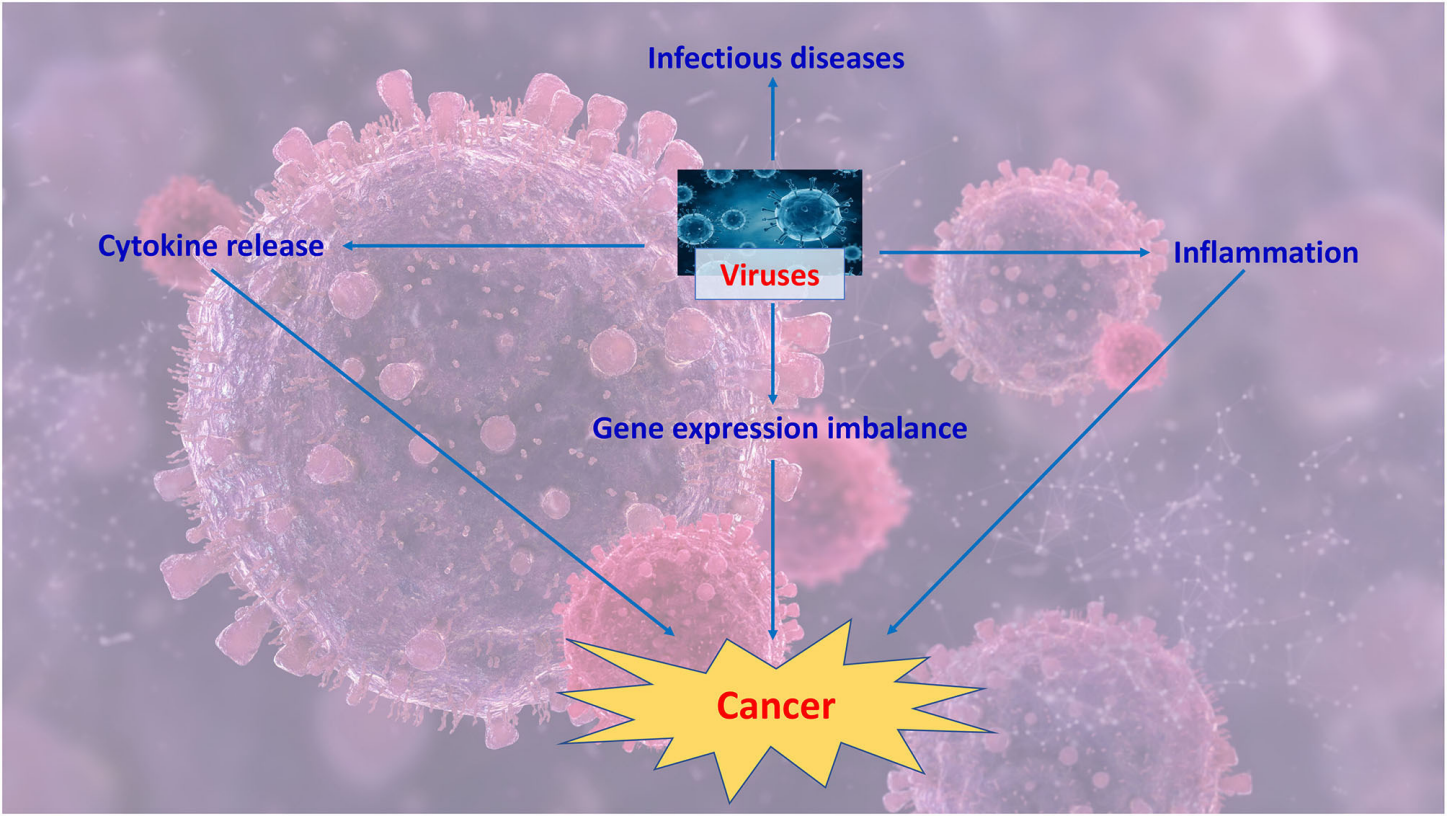
Viruses might contribute to cancer by inducing a prolonged inflammation, dysregulating immune response and unbalancing gene expression.

The article “Advances in CMV Management: A Single Center Real-Life Experience,” by Malagola et al. provides an overview of how the introduction of a recently approved drug improved prognosis of allogenic transplant recipients, which is often compromised by CMV infections in a prolonged immunosuppression setting.

In “The good the bad and the tick,” by Cabezas-Cruz et al. the Authors provide an overview about a large variety of tick-borne parasites, which cause several types of infection, focusing in particular on the Protozoon *Theileria* spp. and the bacterium *Anaplasma phagocitophylum*. The Authors illustrate that these pathogens contribute to malignant transformation by unbalancing epigenetic regulation in the host cells, which is in contrast with DNA genetic modifications often observed in virally-driven cancers, thus suggesting that a plethora of different mechanisms can be used by different types of pathogens to dysregulate the host cells and lead to cancer.

However, microbes are not always pathogenic and “good” microbes are crucial for our well-being. This important aspect is highlighted by two articles of this Research Topic. In “The Recombinant Protein Based on *Trypanosoma cruzi* P21 interacts with CXCR4 receptor and abrogates the invasive phenotype of human breast cancer cells,” by Borges et al. it is illustrated how the recombinant p21 protein of *Trypanosoma cruzi* may play a protective role against breast cancer, by downregulating CXCR4 gene expression, and leading to its internalization, thus preventing migration, invasion, and progression in MDA-MB-231 cells. This article is a very good example of how, despite many pathogens' products playing a role in driving malignant transformation, pathogens' proteins can also be used to our own advantage to fight cancer.

“Fine Particulate Matter Exposure Alters Pulmonary Microbiota Composition and Aggravates *Pneumococcus*-Induced Lung Pathogenesis,” by Chen et al. provides an example of how microbiota play a protective role for us as their dysregulation is associated with diseases as pneumonia. In this study, the Authors highlight how exposure to fine particulate matter (PM) with aerodynamic diameter ≤ 2.5 μm (PM_2.5_) is closely correlated with respiratory diseases, which seems to be linked to microbiota dysbiosis, thus promoting disease progression.

On behalf of all topic-Editors, I would like to thank all the Authors for their invaluable contribution to this Research Topic, which has broadened our knowledge and understanding of the Molecular mechanisms of pathogen-driven infectious and neoplastic diseases.

## Author Contributions

GDF wrote the editorial and embedded comments of other topic Editors. All authors contributed to the article and approved the submitted version.

## Conflict of Interest

The authors declare that the research was conducted in the absence of any commercial or financial relationships that could be construed as a potential conflict of interest.

